# Changes of the Gastric Mucosal Microbiome Associated With Histological Stages of Gastric Carcinogenesis

**DOI:** 10.3389/fmicb.2020.00997

**Published:** 2020-05-29

**Authors:** Zikai Wang, Xuefeng Gao, Ranran Zeng, Qiong Wu, Huaibo Sun, Wenming Wu, Xiaomei Zhang, Gang Sun, Bin Yan, Lili Wu, Rongrong Ren, Mingzhou Guo, Lihua Peng, Yunsheng Yang

**Affiliations:** ^1^Department of Gastroenterology and Hepatology, The First Medical Centre, Chinese PLA General Hospital, Beijing, China; ^2^Department of Hematology-Oncology, International Cancer Center, Shenzhen University General Hospital, Shenzhen University Health Science Center, Shenzhen, China; ^3^Institute of Soil Science, Chinese Academy of Sciences, Nanjing, China

**Keywords:** gastric microbiota, gastric cancer, intraepithelial neoplasia, intestinal metaplasia, chronic gastritis

## Abstract

The changes of gastric microbiome across stages of neoplastic progression remain poorly understood, especially for intraepithelial neoplasia (IN) which has been recognized as a phenotypic bridge between atrophic/intestinal metaplastic lesions and invasive cancer. The gastric microbiota was investigated in 30 healthy controls (HC), 21 non-atrophic chronic gastritis (CG), 27 gastric intestinal metaplasia (IM), 25 IN, and 29 gastric cancer (GC) patients by 16S rRNA gene profiling. The bacterial diversity, and abundances of phyla Armatimonadetes, Chloroflexi, Elusimicrobia, Nitrospirae, Planctomycetes, Verrucomicrobia, and WS3 reduced progressively from CG, through IM, IN to GC. Actinobacteria, Bacteriodes, Firmicutes, Fusobacteria, SR1, and TM7 were enriched in the IN and GC. At the community level, the proportions of Gram-positive and anaerobic bacteria increased in the IN and GC compared to other histological types, whereas the aerobic and facultatively anaerobic bacteria taxa were significantly reduced in GC. Remarkable changes in the gastric microbiota functions were detected after the formation of IN. The reduced nitrite-oxidizing phylum Nitrospirae together with a decreased nitrate/nitrite reductase functions indicated nitrate accumulation during neoplastic progression. We constructed a random forest model, which had a very high accuracy (AUC > 0.95) in predicating the histological types with as low as five gastric bacterial taxa. In summary, the changing patterns of the gastric microbiota composition and function are highly indicative of stages of neoplastic progression.

## Introduction

The development of gastric cancer (GC) is a multistep process affected by multiple factors that involve a complex of molecule networks, which remains largely unclear (Ajani et al., 2017). Normal gastric mucosa undergoes the progressive histologic stages from chronic gastritis (CG), atrophy, intestinal metaplasia (IM), intraepithelial neoplasia (IN), and eventually to GC (Rugge et al., 2016). Given the capacities of inducing inflammation and progressively degrading gastric epithelium, *Helicobacter pylori* has been recognized as group I carcinogen for GC (Plottel and Blaser, 2011). Colonization of *H. pylori* has been correlated with a reduction in gastric microbiota diversity and alterations in bacterial composition (Li et al., 2017; Ren et al., 2018; Zhao et al., 2019). Eradication of *H. pylori* was shown to restore the gastric bacterial diversity and reduce the risk of GC as well as prevent metachronous GC (Li et al., 2017; Choi et al., 2018). However, there were also strong evidences showing that *H. pylori* eradication does not completely prevent persistent inflammation of the gastric mucosa and gastric carcinoma development, suggesting that GC development does not depend completely upon *H. pylori* infection (Cheung et al., 2018). This hypothesis is supported by animal studies showing that transgenic hypergastrinemic insulin-gastrin (INS-GAS) mice are more susceptible to *H. pylori*-induced GC than germ-free mice (Lofgren et al., 2011; Lertpiriyapong et al., 2014). In addition to *H. pylori* infection and antibiotics, treatment with proton pump inhibitors (PPIs) was also shown to perturbate the composition of gastric microbiota, and particularly increase the level of *Streptococcus* (Amir et al., 2014; Sterbini et al., 2016).

To date, the overall knowledge on the roles of non-*H. pylori* gastric microbes in the gastric neoplastic progression is still limited. A few studies have investigated the gastric microbiota profile in different gastric histologic types, however no consistency changes in the microbial richness, diversity, or composition (Aviles-Jimenez et al., 2014; Eun et al., 2014; Wang et al., 2016; Coker et al., 2018; Ferreira et al., 2018). Aviles-Jimenez et al. (2014) demonstrated a progressive shift in gastric microbiota profile from non-atrophic gastritis to IM to GC, which suggested a reduction in Porphyromonas, Neisseria, TM7, and *Streptococcus sinensis*, as well as an increase in *Lactobacillus coleohominis* and Lachnospiraceae might favor gastric tumorigenesis. Using pyrosequencing, Eun et al. (2014) showed that the Bacilli class and Streptococcaceae family were enriched in GC patients, while the Epsilonproteobacteria class and Helicobacteraceae family decreased significantly compared to those with CG and IM. A recent study by Coker et al. (2018) demonstrated that phylum Fusobacteria, and genera *Dialister*, *Mogibacterium*, and *Peptostreptococcus* were also more abundant in GC than in superficial gastritis, atrophic gastritis, and IM. A more recent study by Ferreira et al. (2018) revealed that gastric microbiota in GC patients had a decreased diversity, reduced *Helicobacter* abundance, and an enrichment in genera *Citrobacter*, *Clostridium*, *Lactobacillus*, *Achromobacter*, and *Rhodococcus* compared with CG. The discrepancy among these studies may partially due to small numbers of subjects, different sequencing techniques, and different gastric histological samples. Of particular note is that the gastric microbiota profile in IN has been seldom studied.

The present study accessed the changes in the gastric microbiota composition and function and their association with the progressive histological types along gastric neoplastic progression—from CG through IM, low-grade IN, high-grade IN to GC. We also explored the use of gastric bacterial taxa as biomarkers to classify the different histological stages of gastric tumorigenesis.

## Materials and Methods

### Study Population

This study was approved by the Ethics Committee of the Chinese PLA General Hospital, Beijing, China, and was registered at the WHO ICTRP (ID: ChiCTR-OCC-12002573). Written informed consent was obtained from all the participants. The study cohort was recruited from May 2012 to December 2016. Health individuals were recruited from a physical examination population; CG, IM, IN, and GC patients were enrolled after diagnosis through endoscopic and histopathological examinations by experienced endoscopists and histologists. The demographics, medical history, medication, and blood tests (e.g., fasting glucose, cholesterol, triglyceride, etc.) were assessed for all subjects.

The inclusion criteria were: (1) adult man or women; (2) Han nationality from northern areas of China; (3) to provide signed and dated informed consent; and (4) to provide gastric mucosal biopsy samples. Furthermore, the exclusion criteria were: (1) taking antibiotics, PPIs, probiotics, prebiotics, chemotherapeutic drugs, and any other drugs affecting gastrointestinal microbiota within the last month; (2) acute or chronic pulmonary, cardiovascular, hepatic, or renal disorders; (3) positive test for human immunodeficiency virus, hepatitis B or C virus; (4) history of major surgery; and (5) pregnant or lactating.

### Endoscopic Mucosal Tissue Sampling and Histologic Examination

All participants were fasting and took no drugs affecting gastrointestinal microbiota when receiving gastroscopy. Both the endoscopic and histological examinations were performed for all subjects according to internationally accepted criteria (Dixon, 2002; Fang et al., 2007). Gastric mucosal biopsy samples of 1–2 mm were obtained using standard gastroscopic forceps. Biopsy for histologic examination was performed based on the disease condition and as needed [healthy controls (HC): one biopsy sample; CG and IM: two biopsies from gastric antrum, corpus or angle; GC: three biopsies from the definite and suspected cancerous lesions]. The gastric biopsy samples for histologic examination were fixed in 10% formalin and placed in separate vials which were labeled according to their topographic site (antrum, angularis incisura, or corpus). Additional mucosal biopsy specimens were taken for microbial analysis (HC, CG, and IM: two biopsies from the gastric antrum and body; IN: two gastric mucosal biopsies from the gastric antrum and body and one IN tissue; GC: two gastric mucosal biopsies from the gastric antrum and body and one tumorous tissue). The biopsy specimens for microbial analysis were frozen in liquid nitrogen immediately and transferred to the laboratory and stored at −80°C until DNA extraction.

The histological evaluation of IN was based on the specimen after endoscopic submucosal dissection. HC subject had a negative ^13^C urea breath test (UBT) result, no digestive symptoms, and was further confirmed with normal superficial foveolar and deep glandular compartments via histologic examination. Non-atrophic CG was confirmed according to the density and the infiltrating depth of chronic inflammatory cells in the mucosa, without the reduction of gastric glands proper at each biopsy site. The diagnosis of IM was confirmed by the presence of differentiated epithelium in the area of the glands and superficial epithelium at any biopsy site. The diagnosis and classification of IN was determined by revised Vienna classification system (Dixon, 2002). GC was confirmed with gastric adenocarcinoma, and was divided into diffuse, intestinal, and mixed types according to Lauren classification.

### DNA Extraction and Polymerase Chain Reaction Amplification

Total DNA was extracted from gastric tissue biopsies by using the QIAamp DNA Mini Kit (Qiagen, Valencia, CA, United States) combined with the bead-beating method. After agitation by the bead beater (Fast Prep FP120; Qbiogene, Carlsbad, CA, United States), bacterial DNA was extracted. The initial DNA concentration of each sample is available in Supplementary Table S1. The DNA concentration was adjusted to 50 ng/μl for each sample and stored at –80°C prior to sequencing.

The V4 region of the 16S rRNA gene was sequenced using universal primers targeting most bacteria (F: 5′-GTGCCAGCMGCCGCGGTAA-3′, R: 5′- GGACTACHVGGGTWTCTAAT -3′) with a 6-bp barcode unique to each sample for the paired primer. Thermal cycling conditions were consisted of initial denaturation at 98°C for 1 min, followed by 35 cycles of denaturation at 98°C for 10 s, annealing at 50°C for 30 s, and elongation at 72°C for 60 s, with the final extension at 72°C for 5 min. The single amplifications were performed in 25 μl reactions with 50 ng template DNA. Normalized equimolar concentrations of polymerase chain reaction (PCR) products were pooled and sequenced using the Illumina MiSeq PE300 platform (Illumina, San Diego, CA, United States).

### 16S rRNA Gene Sequencing Data Processing

Demultiplexing and quality filtering of sequencing data was performed using *split_libraries_fastq* in QIIME 1.9.1 with the standard parameters (Caporaso et al., 2010), which yielded 10,133,704 sequences with an average of 32,689 sequences per sample. Sequences were then clustered into OTUs based on to 97% similarity by using the QIIME *pick_closed_reference_otus* function. Representative sequences for each OTU were aligned against the non-redundant Greengenes database (version 13.8). Singletons were removed, and the data were rarefied to 9400 sequences per sample to control for variations in sequencing efforts. The rarefaction analysis suggests that the sequencing depth could recover most of the diversity of the gastric bacterial community (Supplementary Figure S1).

### Functional Prediction of Gastric Bacterial Microbiota

Functional predictions were performed using Phylogenetic investigation of communities by reconstruction of unobserved states (PICRUSt) (Langille et al., 2013). The obtained OTU table was normalized by 16S rRNA copy number and metagenomes were predicted with Kyoto Encyclopedia of Genes and Genomes (KEGG) catalog (Kanehisa and Goto, 2000). Predicted functional genes were classified into clusters of orthologous groups (COG) of proteins and into KEGG orthology (KO). BugBase (Ward et al., 2017) was employed to predict organism-level coverage of functional pathways and biologically interpretable phenotypes. The relative abundances of six phenotypic categories, including Gram staining, oxygen tolerance, ability to form biofilms, mobile element content, pathogenicity, and oxidative stress tolerance, were compared across the disease stages.

### Statistical Analysis

The Calypso (version 8.84) (Zakrzewski et al., 2017) was applied for the statistical analysis. The read counts were normalized with total sum normalization, and taxa that have less than 0.01% relative abundance across all samples were excluded from the following analysis. Microbial alpha diversity was measured in the number of OTUs, Shannon index, Chao1 index, and phylogenetic diversity (Supplementary Table S2). Principal coordinate analysis (PCoA) and non-metric multidimensional scaling (NMDS) was performed to determine differences between microbial communities using Bray–Curtis dissimilarity and weighted and unweighted UniFrac distance matrices. Taxa abundances and alpha diversity indices were compared by one-way analysis of variance (ANOVA), followed by pairwise *t*-test. The linear discriminant analysis (LDA) effect size method (LEfSe) (Segata et al., 2011) was used to determine the PICRUSt-predicted functions that associated with different gastric histological types. Differences in the BugBase’s organism-level microbiome phenotypes were accessed using Wilcoxon rank test, with *p* values corrected using the Benjamini-Hochberg method.

### Random Forest Classifier

A random forest model was constructed using the using the R package randomForest (White et al., 2009). The whole dataset was randomly divided into training and testing datasets, with each consisting of 50% of the samples. The tuneRF function in randomForest was performed to assess the optimal number of variables at a node split (i.e., the *mtry* parameter). Prediction performance of the model was evaluated by out-of-bag (OOB) error and area under the receiver operating characteristic (ROC) curve (AUC). The R implementation of random forest algorithm is available at Supplementary Code S1.

## Results

### Demographic Characteristics

Finally, 30 HC, 21 CG, 27 IM, 25 IN (10 low-grade and 15 high-grade), and 29 GC (11 cardia and 18 non-cardia adenocarcinoma) patients were enrolled (Supplementary Table S3). In total, 310 eligible gastric mucosal biopsy samples for microbial analysis were included. Age, sex, and BMI were not significantly different across the disease stages (*p* > 0.05). The levels of fasting blood glucose, total cholesterol, and triglyceride fell in the normal range for all participants.

### General Composition of Gastric Mucosal-Associated Bacterial Microbiota

A total of 73 bacterial phyla were identified from all gastric biopsy samples, and the top six most abundant phyla were Actinobacteria, Bacteroidetes, Cyanobacteria, Firmicutes, Fusobacteria, and Proteobacteria (Supplementary Figure S2A); *Acinetobacter*, *Aquicola*, *Haemophilus*, *Halomonas*, *Helicobacter*, *Lactobacillus*, *Prevotella*, *Shewanella*, *Sphingomonas*, *Streptococcus*, and *Veillonella* were the most predominant genera in the stomach (Supplementary Figure S2B).

To access the microbiota characteristics at different anatomical sites of gastric mucosa, we compared pairs of antrum and body biopsy samples for each disease stage. No significant difference in the microbiota alpha diversity was identified between the paired samples of gastric antrum and body mucosa in each disease stage, further indicating a great similarity between these two anatomical sites in heath and disease status (Supplementary Figure S3). At the OTU level, the inter-group differences in gastric microbiota composition of antrum and body were not significant compared with that of the intra-group (Supplementary Figure S4). Moreover, the bacterial community composition was similar between the lesion surface and the non-lesion mucosa of gastric antrum and body in IN or GC. All analysis henceforward did not differentiate based on sample types.

### Mucosal Bacteria Changes Are Associated With Gastric Neoplastic Progression

**FIGURE 1:**
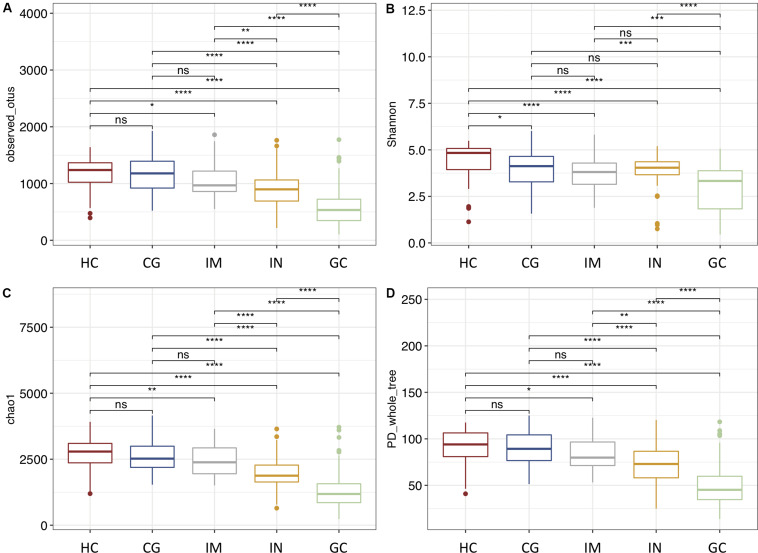
The alpha diversity of gastric microbiota reduces from HC through CG, IM, IN to GC. Diversity estimates were obtained from OTU richness and evenness by using **(A)** observed number OTUs, **(B)** Shannon index, **(C)** Chao1 index, and **(D)** PD whole tree. Statistically significant differences in alpha diversities were analyzed by *t*-test and annotated as *p < 0.05, **p < 0.01, ***p < 0.001, ****p < 0.0001.

**FIGURE 2:**
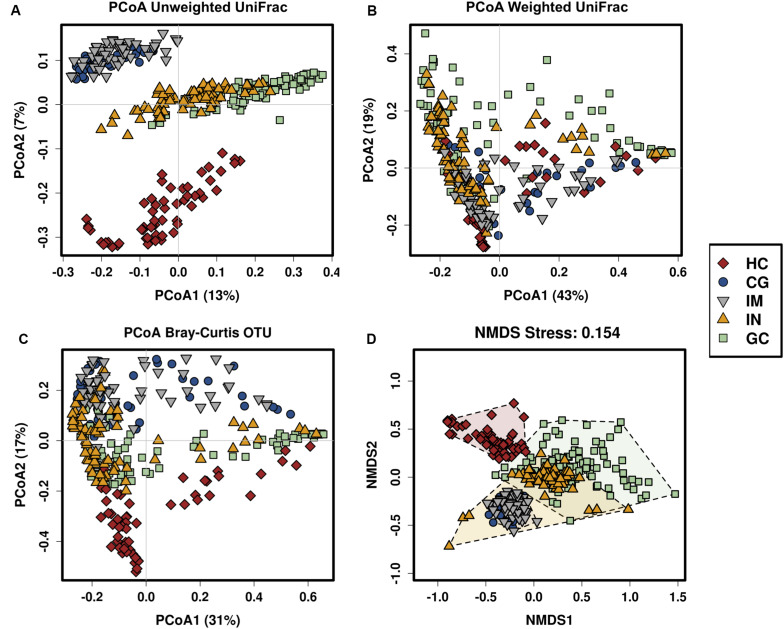
The gastric microbiota profile differs in HC and patients with CG, IM, IN, and GC. Principal coordinate analysis (PCoA) of **(A)** unweighted UniFrac distance matrix, **(B)** weighted UniFrac distance matrix, **(C)** Bray–Curtis distance matrix, and **(D)** non-metric multidimensional scaling (NMDS) based on the OTUs in which samples are colored and shaped by phenotype of the gastric mucosa.

To identify trajectory of the mucosal bacteria changes alongside gastric neoplastic progression, we compared the richness, diversity, and composition of gastric bacterial community among the disease stages. Gastric microbial richness and alpha diversity dropped progressively from HC, CG, IM, IN to GC, and significantly lower in the IN and GC patients (Figures 1A–D). Moreover, the alpha diversity was neither significantly different between the patients with low- and high-degrade gastric IN, nor between gastric cardia and non-cardia cancer. Beta diversity analysis with PCoA and NMDS based on the OTU level revealed a pattern in which the samples were assigned into four separate groups (Figures 2A–D). A separate clustering was obtained between HC and all patient samples, indicating dysbiosis of the gastric microbiota associated with disease. The clusters of IM and CG were close to each other, suggesting a similar gastric microbiota profile. Furthermore, the gastric microbiota of IN showed similarity to GC. Since age has been identified as a risk factor for GC, we addressed whether the bacterial profile was distinguishable between old and young individuals for each disease stage. Results of PCoA based on OTUs indicated that age could not differentiate the microbiota profile among the disease stages (Supplementary Figure S5).

**FIGURE 3:**
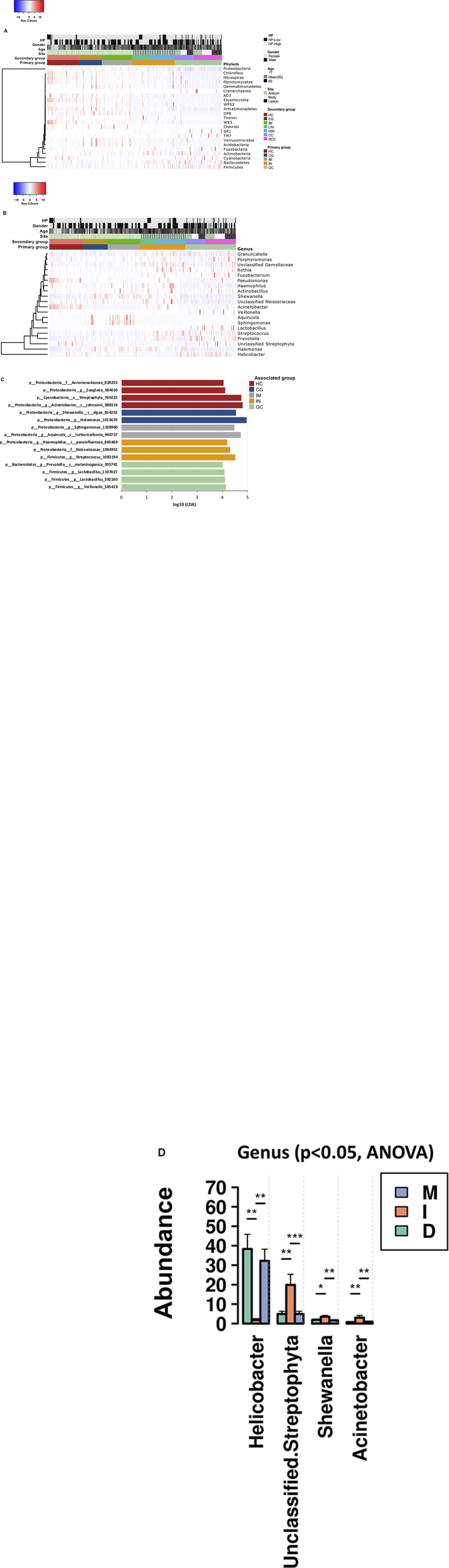
Changes in the gastric microbiota from HC, through CG, IM, IN to GC. Heatmaps of significantly different **(A)** phyla and **(B)** genera across the disease stages. Significance was determined by one-way ANOVA with *p* < 0.05 (Supplementary Table S4). **(C)** Association of specific bacteria taxa with different disease stages was identified by LEfSe with Kruskal–Wallis test *p* < 0.05 and log^10^ LDA score > 3.0. **(D)** Four genera were differentially abundant between subtypes of GC. Pair-wise comparisons are done by *t*-test and annotated as *p < 0.05, **p < 0.01, ***p < 0.001.

Relative abundances of multiple phyla were found to be reduced from HC, through CG, IM, IN to GC, including Armatimonadetes, Chloroflexi, Elusimicrobia, Nitrospirae, Planctomycetes, Verrucomicrobia, and WS3; Acidobacteria, Gemmatimonadetes, Proteobacteria, and Verrucomicrobia were found enriched in CG and IM patients; Actinobacteria, Bacteriodes, Firmicutes, Fusobacteria, SR1, and TM7 were more abundant in the IN and GC compared to other histological types (Figure 3A and Supplementary Table S4). At the genus level, there were 284 genera significantly different (ANOVA *p*-value < 0.05) across the disease stages (Supplementary Table S4). For the top 20 most abundant genera (Figure 3B), the levels of *Halomonas* and *Shewanella* were significantly higher in biopsy samples of the patients (especially in CG and IM), whereas *Acinetobacter* and *Pseudomonas* were more abundant in HC (Supplementary Table S4). Both CG and IM had higher abundances of *Aquincola* and *Sphingomonas* than other disease stages. The relative abundances of genera *Granulicatella*, *Porphyromonas*, unclassified Gemellaceae, *Rothia*, and *Fusobacterium* were higher in patients with IN; *Helicobacter* and *Lactobacillus* were significantly enriched in GC; and increased levels of *Streptococcus*, *Prevotella*, and *Veillonella* were observed in both IN and GC.

To identify the most relevant taxa responsible for the differences among the disease stages, we performed LEfSe analysis based on the OTUs (Figure 3C). In GC, an enrichment of three Firmicutes taxa was observed, including a taxon of *Veillonella* and two taxa of *Lactobacillus*. Species belonging to *Streptococcus* genus, Neisseriaceae family, and the *Haemophilus parainfluenzae* were significantly more abundant in IN. Among the three subtypes of GC, the intestinal-type showed a significant lower abundance of *Helicobacter*, and higher abundances of *Acinetobacter*, *Shewanella*, and unclassified Streptophyta than the diffuse and mixed types (Figure 3D). The IM was featured by a higher abundance of *Aquincola tertiaricarbonis* and a taxon of *Sphingomonas* genus. Patients with CG had an increased level of *Shewanella algae* and a taxon of *Halomonas* genus, both belonging to the Proteobacteria phylum. The reprehensive bacteria in HC were three Proteobacteria taxa including *Acinetobacter johnsonii*, a taxon of *Zoogloea* genus and a taxon of Aeromonadaceae family, and a taxon of Streptophyta order (Cyanobacteria phylum).

By using BugBase, we predicted organism-level microbiome phenotypes including Gram staining, oxygen tolerance, and pathogenic potential, and compared their relative abundances among the disease stages (Supplementary Figure S6 and Supplementary Table S5). The stress-tolerant microorganisms were predicted to be decreased in CG and IM. Compared with other histological types, patients with IN and GC have a significantly higher level of Gram-positive and a lower level of Gram-negative bacteria, respectively. Moreover, anaerobic bacteria were enriched in IN and GC patients, mainly due to an increase in Bacteroidetes and Firmicutes phyla. Aerobic and facultatively anaerobic bacteria, as well as biofilm forming, and potentially pathogenetic species were significantly attenuated in patients with GC, which attributed to the decreased abundance of Proteobacteria.

### A Portion of GC and IN Cases Present Decreased Abundance of *H. pylori*

All enrolled participants were tested for *H. pylori* infection by the ^13^C UBT. The *H. pylori* infection rate was 28.6% (6/21) in CG, 33.3% (9/27) in IM, 60% (15/25) in IN, and 58.6% (17/29) in GC patients, and none of HC was positive for *H. pylori* (Supplementary Table S6). By using the 16S rRNA sequencing, *H. pylori* sequences were detected in all mucosa samples in the CG, IM, IN, and GC, and only three samples from two healthy individuals were negative. The average relative abundance of *H. pylori* was the highest in GC (Supplementary Figure S7A).

Based on the relative abundance of *H. pylori*, we stratified the samples into *H. pylori*-low (relative abundance < 0.01) and *H. pylori*-high (relative abundance = 0.01) according to previous studies (Kim et al., 2015; Zhao et al., 2019). PCoA showed that the overall composition of gastric microbiota was not able to be separated between these two groups (Supplementary Figure S7B). The *H. pylori*-low samples were mainly found in the HC, IN, and GC. Overall, these results show that *H. pylori* is prevalent in both health controls and patients with gastric disorders, even in *H. pylori* negative individuals examined by the conventional clinical tests. The presence of *H. pylori* was linked to mucosal microbiome dysbiosis in gastric lesions, featured by decreased biodiversity. It is worth noting that patients with intestinal type of GC and IN had a reduced abundance of *H. pylori*.

### Gastric Microbiota Is Predictive of Stages of Gastric Carcinogenesis

**FIGURE 4:**
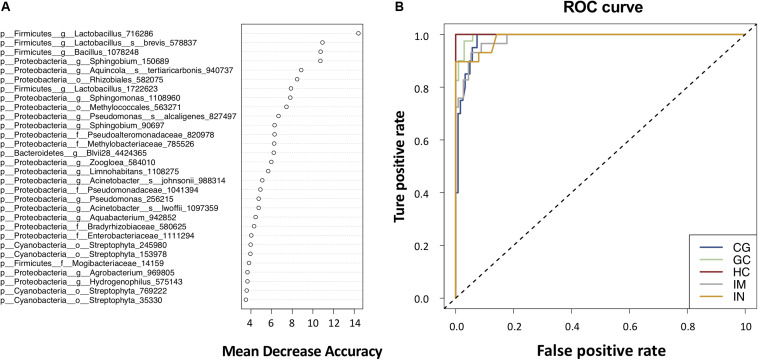
Gastric bacterial biomarkers for classifying histological types. **(A)** The random forest classifier identified 30 bacterial taxa that are most discriminatory among the disease stages in descending order. Each OTU was assigned an importance score (mean decrease accuracy). **(B)** ROC curves analysis to evaluate the discriminatory potential of gastric bacteria in identifying different.

To illustrate the potential of the gastric mucosal bacterialmicrobiota as diagnostic biomarkers, we constructed a random forestmodel based on the top 100 most significant OTUs for distinguishing between gastric histological types. The model had an OOB error rate of 7.74%; the classification error was 3.13, 23.81, 16.00, 2.17, and 4.55% for HC, CG, IM, IN, and GC, respectively (Supplementary Table S7). The classification error rate was higher in CG and IM compared to the rest disease stages, further indicating a higher similarity between the gastric microbiome of CG and IM. Thirty significantly discriminatory OTUs were identified and were arranged in rank order of their contribution to the classification accuracy (Figure 4A). The model showed an excellent performance in predicting the gastric histological types against the test data, yielding an AUC of 1.0, 0.98, 0.98, 0.99, and 1.0 for HC, CG, IM, IN, and GC, respectively (Figure 4B). By reducing the number of features through 30, 10 to 5 OTUs (g__*Sphingobium*_150689, g__*Lactobacillus*_716286, s__*Aquincola_tertiaricarbonis*_940737, g__*Bacillus*_1078248, and s__*Acinetobacter_johnsonii*_988314; see Supplementary Code S1), only a minor decrease in accuracy was observed (Supplementary Table S7).

### Gastric Microbiome Functional Capacity Changes Alongside Gastric Neoplastic Progression

**FIGURE 5:**
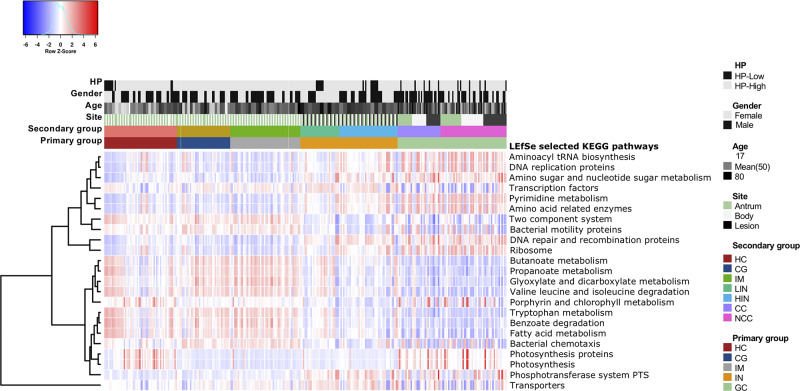
Functional dysbiosis in the gastric microbiota is associated with the progression of gastric carcinoma. Heatmap of differential expression in functional metabolic pathways across stages of gastric carcinogenesis. The KEGG pathways significantly enriched in each disease stage were identified using LEfSe with Kruskal–Wallis test *p* < 0.05 and log_10_ LDA score > 3.0.

We applied PCIRUSt to infer the metabolic functions based on the microbial community profiles obtained from the 16S rRNA gene sequences. Overall, the bacterial communities present in patients with GC and IN were distinct from other disease stages (Supplementary Figure S8). The predicted KEGG pathways significantly enriched in GC included aminoacyl-tRNA biosynthesis, amino acid related enzymes, amino sugar and nucleotide sugar metabolism, DNA repair and recombination proteins, DNA replication proteins, pyrimidine metabolism, photosynthesis, and ribosome were observed (Figure 5 and Supplementary Table S8). Conversely, pathways involved in bacterial chemotaxis, bacterial motility proteins, benzoate degradation, butanoate metabolism, fatty acid metabolism, glyoxylate and dicarboxylate metabolism, valine, leucine, and isoleucine degradation, propanoate metabolism, tryptophan metabolism, and two-component system significantly decreased in GC and IN.

In the previous section, we observed that Nitrospirae, a nitrite-oxidizing phylum decreased progressively from HC, through CG, IM, IN to GC, suggesting a potential alteration in the nitrogen cycle alongside cancer progression. Since exposure to high concentrations of nitrate, nitrite, and N-nitroso (NO) compounds may contribute to gastric malignant transformation (Lucker et al., 2010; Spieck and Lipski, 2011), we next accessed the microbial functions involved in those metabolic reactions. When compared with HC, the nitrate reductase function decreased across the disease stages (Supplementary Figures S9A,B and Supplementary Table S8), and remarkably lower in IN and GC (e.g., COG0600, COG0715, KO0371, KO2049, and KO2050). Moreover, the nitrite reductase function was found to be reduced in IN and GC (e.g., COG2146, K00362, and K00363).

## Discussion

In this study, the changes of gastric mucosal bacterial microbiota were found to be strongly associated with stages of gastric carcinogenesis, including CG, IM, IN, and GC. Our data showed that the diversity and richness of gastric microbiome compositions reduced progressively across histological stages of gastric tumorigenesis. Consistent with previous studies (Bik et al., 2006; Yu et al., 2017), no significant difference was identified in the microbiome between biopsies of the gastric antrum and body. Taxonomical composition differences between non-malignant and tumor tissues were not detected, which was not in line with the findings of another study (Yu et al., 2017). Due to the limited phylogenetic resolution and lower sensitivity of 16S amplicon sequencing, the use of whole genome shotgun sequencing is needed to address whether the microbial profile was different among different anatomical sites and tissue types (e.g., non-cancerous and tumor lesions).

The next generation sequencing-based methods such as 16S rRNA and metagenomic sequencing were shown to be more sensitive in detecting mucosa microbes, and could identify *H. pylori* in conventionally *H. pylori*-negative cases (Kim et al., 2015; Zhao et al., 2019). In our study, the *H. pylori* sequences were detected in nearly all samples, and their relative abundances were similar regardless of the different histological stages of gastric neoplastic progression. In particular, no clear association was found between the age and the relative abundances of *H. pylori*. Some studies demonstrated that *H. pylori* infection changes the composition of gastric microbiota (Sterbini et al., 2016; Noto et al., 2019). However, contradictory findings argued that *H. pylori* or its virulence factors likely have limited influence on the gastric microbiota changes (Bik et al., 2006; Yang et al., 2016). In our study, the significant differences in gastric microbial diversity and composition among the various stages of gastric carcinogenesis were largely independent of *H. pylori* infection. Early studies showed that the abundance of *Helicobacter* reduced in stomach of GC (Kang et al., 2006; Ferreira et al., 2018). Our data revealed that the level of *H. pylori* was particularly decreased in the intestinal-type GC when compared to the disuse type. Nevertheless, a large number of cases are needed to further investigate if *H. pylori* infection was associated with some subtypes of GC.

The alpha diversity was not significantly different between CG and IM patients, and the composition of their microbiome was also similar featured by higher relative abundances of phyla Acidobacteria, Gemmatimonadetes, and Verrucomicrobia. This result indicates that the gastric microbial changes mildly alongside the progression from CG to IM, albeit their histological features are apparently distinct. Certain genetic and epigenetic alterations have been demonstrated in gastric IM (Leung and Sung, 2002); nevertheless, the associated functional changes of gastric mucosa have not been confirmed. Our results suggest that the gastric environment factors that determine the microbiota composition may be similar between CG and IM.

The gastric mucosal microbiome diversity in IN reduced dramatically compared to HC, CG, and IM. Compositionally, multiple phyla were enriched in patients with gastric IN, including Actinobacteria, Bacteroidete, Firmicutess, and Fusobacteria. Furthermore, the gastric microbial community was similar between low- and high-grade IN. Thus, the changes in gastric microbiome maybe preceded the onset of IN, which became tremendous during the progression from IM to IN. A recent study showed that the microbial richness and diversity in GC were different from that of CG and IM, which also confirmed the presence of microbial dysbiosis in gastric carcinogenesis (Coker et al., 2017). Our analysis showed that the microbial composition had already changed at the phylum level in patients with gastric precancerous lesions, and some changes maintained through cancer procession. For example, the higher relative abundance of Actinobacteria, Bacteroidetes, Firmicutes, and Fusobacteria and lower relative abundance of Acidobacteria and Proteobacteria were found in both IN and GC patients. These observations were partly consistent with previous studies (Bik et al., 2006; Andersson et al., 2008; Frey-Klett et al., 2011). At the community level, Gram-positive and anaerobic bacteria were found with higher abundances in patients with IN and GC; facultatively anaerobic and biofilm forming bacteria were significantly attenuated in patients with GC compared to other disease stages, mainly attributed to the reduced abundances of some Proteobacteria species. Several taxa associated with gastrointestinal cancers have been identified in the patient samples from our study. The halophilic genera *Halomonas* and *Shewanella*, which were recently found associated with rectal cancer (Flemer et al., 2017), were enriched in the host stomach across stages of gastric carcinogenesis. As a ubiquitous organism, *Shewanella* are opportunistic pathogens that associated with gastrointestinal infection (Wang et al., 2013). However, the specific strains and functions of these enriched halophilic bacteria in human digestive tract worth to be further investigated in terms of their potential contribution to carcinogenesis.

Until now, few studies have focused on whether new methods of early warning and diagnosis of GC can be established based on gastric microbiota. In the present study, we constructed a random forest classifier as a predictive model for identifying different histological stages of GC based on gastric microbiota profile. This predictive model showed an excellent performance to discriminate the disease stages, suggesting that these significantly associated non-*H. pylori* genera could be used as the potential microbial biomarkers for GC and precancerous lesions. Due to microbiota variation across populations, a larger number of samples from multicenter are necessary to validate the predictive performance of the model-identified biomarkers. Among these potential biomarkers, we noticed a taxon assigned to *A. tertiaricarbonis* that was capable of degrading methyl tert-butyl ether (MTBE). MTBE has been considered as a weak cancer potency given its ability in inducing DNA damage and oxidative base modification (Bogen and Heilman, 2015). Thus, *A. tertiaricarbonis* may play a protective role in gastric carcinogenesis. A taxon assigned to *A. johnsonii* was also found to be important in contributing the accuracy of the random forest model. *A. johnsonii* is the one of the most predominant bacterial species found in foods, which rarely cause human infections. However, animal model studies indicated that some *Acinetobacter* species such as *Acinetobacter lwoffii* were able to induce hypergastrinemia, gastritis, and increase gastric epithelial cell numbers, as observed with *H. pylori* (Zavros et al., 2002). Hence, a further functional examination of *Acinetobacter* spp. isolated from patients with gastric diseases may reveal the role of this non-*H. pylori* organism in gastric carcinogenesis.

The functional compositions of the HC, CG, and IM were similar to each other, but differed significantly from IN and GC patients. This observation implicates that the diversity and composition of gastric microbiota might have shifted in IM, but the functional capacity of the microbiome might not have altered at this stage. We observed a switch toward galactose metabolism, purine metabolism, and pyrimidine metabolism in IN and GC compared to other disease stages. Notably, the functional content associated with bacterial toxins was found overexpressed in the GC patients. Specific toxins released by dysbiotic microbiota that have the capacity to directly induce DNA damage and/or indirectly induce chronic inflammation thus modulating tumorigenesis. In addition, our study inferred that functional categories corresponding to DNA damage and repair process such as nucleotide excision repair, mismatch repair, DNA repair and recombination proteins, and DNA replication were enriched in GC. This may indicate the activity of gastric microbiome in restoring its homeostasis.

Nitrate and nitrite educing commensal bacteria have a symbiotic role in facilitating the nitrogen cycle (Lundberg et al., 2004). Nitrite could derive either directly from food, or by reduction of nitrate via nitrate reductase produced by oral commensal bacteria. Nitrite is further reduced into NO via nitrite reductase produced by gastric commensal bacteria. The nitrite-derived NO were thought to play an important role in host defense and in regulation of gastric mucosal integrity (Petersson et al., 2015). However, nitrate or nitrite was also considered to be harmful due to the potential formation of carcinogenic nitrosamines by reacting with amines under acidic condition in stomach (Jakszyn et al., 2006; Hernandez-Ramirez et al., 2009). Theses bacteria encode nitrite oxidoreductase which oxidizes the formation of nitrate from nitrite (Lucker et al., 2010; Spieck and Lipski, 2011), thereby decreasing the nitrite production. Our data show a decreased abundance of Nitrospirae in IN and GC, suggesting a potential increased level of nitrite in stomach. Moreover, the functional prediction shows a decline in the nitrite reductase in IN and GC, further enhancing the hypothesis of nitrite accumulation participating in malignant transformation.

Certainly, there were several limitations in this study. The gastric microbiota features of atrophic gastritis were not depicted due to a lack of such patients in our cohort, and then IM patients involved in this study were not evaluated by the histological grade of IM. The large sample size microbial studies based on the staging and grading systems of Operative Link for Gastritis Assessment (OLGA)/Operative Link on Gastric Intestinal Metaplasia Assessment (OLGIM) maybe contribute to further characterize the gastric microbiota changes associated with the evolution of atrophy and IM (Rugge and Genta, 2005; Capelle et al., 2010). In addition, 16S rRNA gene sequencing has low phylogenetic power at the species/strain level and poor discriminatory power for some genera.

## Conclusion

The results of our study confirm a shift in the gastric microbial community structure and functions along gastric neoplastic progression. The diversity and composition of gastric mucosa-associated bacterial microbiota change progressively cross stages of gastric carcinogenesis. GC and its precancerous lesions have distinguishable bacterial taxa and functional properties. The constructed predictive model is able to accurately identify the gastric histological type based on a small number of gastric bacteria. A large-scale multicenter study is warranted to identify taxonomic biomarkers with more general applicability in distinguishing stages of gastric carcinogenesis.

## Data Availability Statement

The 16S rRNA gene NGS data generated for this study have been deposited in European Nucleotide Archive (https://www.ebi.ac.uk/ena/submit/sra/#studies) with the accession number of PRJEB26931.

## Ethics Statement

The studies involving human participants were reviewed and approved by the Ethics Committee of the Chinese PLA General Hospital, Beijing, China. The patients/participants provided their written informed consent to participate in this study.

## Author Contributions

YY designed the experiments and revised the manuscript. ZW, RZ, QW, WW, and LW performed the experiments. XG, ZW, and HS analyzed clinical and sequencing data. ZW and XG wrote this manuscript. XZ, GS, BY, RR, MG, and LP contributed to the experimental plan and commented on the manuscript.

## Conflict of Interest

The authors declare that the research was conducted in the absence of any commercial or financial relationships that could be construed as a potential conflict of interest.
