# Supplementary material for: Changes of the Gastric Mucosal Microbiome Associated With Histological Stages of Gastric Carcinogenesis
Source: Front Microbiol. 2020 May 29;11:997. doi: 10.3389/fmicb.2020.00997 (PMC7272699; doi:10.3389/fmicb.2020.00997)
Supplement: Supplementary file 2 [file Presentation_1.pdf]

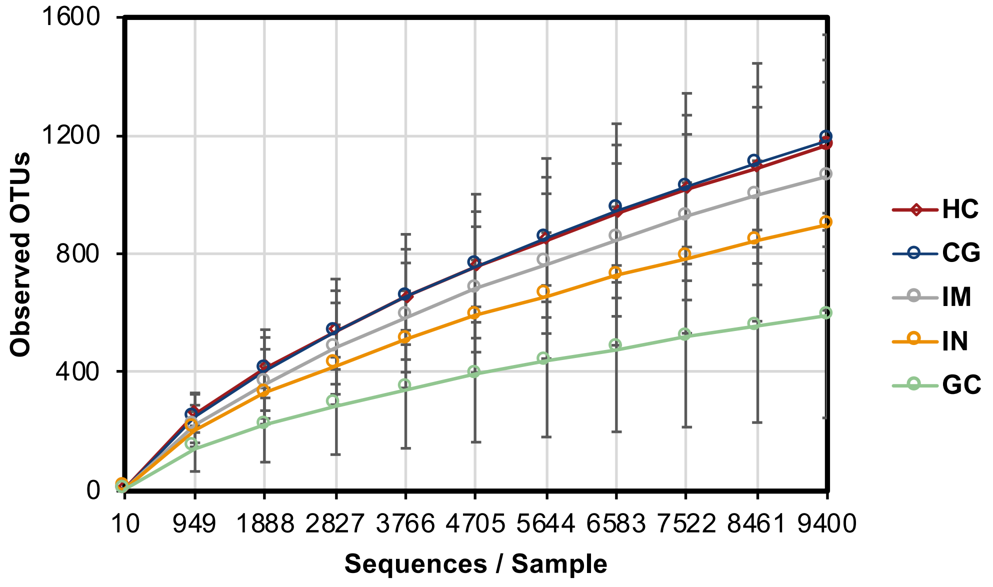


**Supplementary Figure S1.** Rarefaction curves of the numbers of observed OTUs in different sample groups.

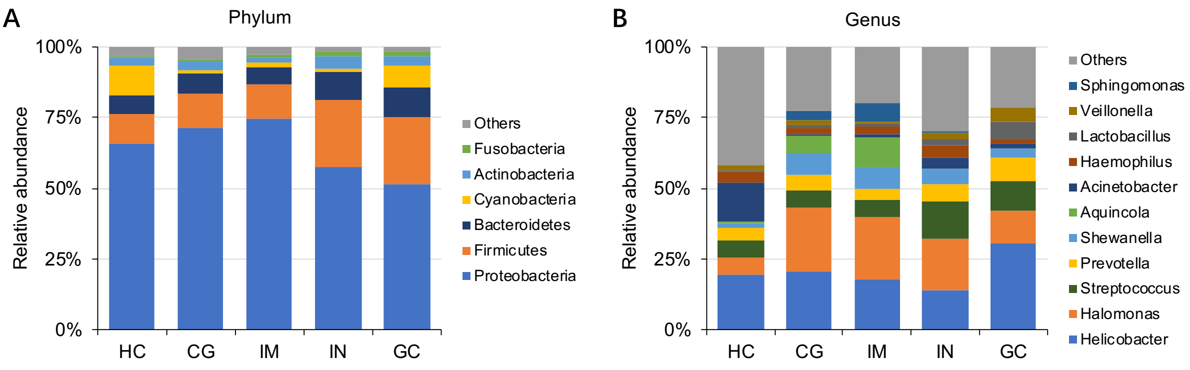


**Supplementary Figure S2**. The general compositional characteristics of gastric bacterial microbiota in HC and patients with CG, IM, IN and GC. Relative abundance of the most abundant (A) phyla and (B) genera in each sample group. HC: healthy controls; CG: chronic gastritis; IM: intestinal metaplasia; IN: intraepithelial neoplasia; GC: gastric cancer.

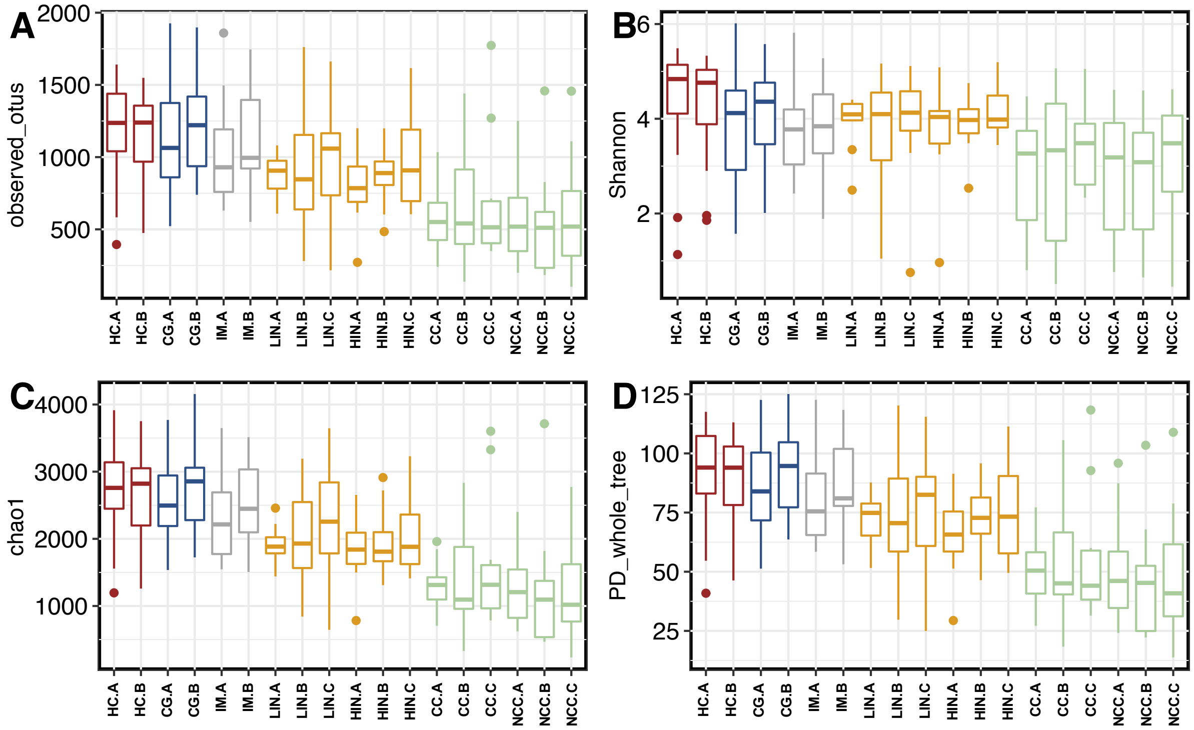


**Supplementary Figure S3.** Alpha diversity of gastric antrum, body and lesion surface biopsies from HC, CG, IM, LIN, HIN, CC and NCC groups. Boxplots of **(A)** observed OTUs, **(B)** Shannon index, **(C)** chao1 index, and **(D)** PD whole tree among different sample groups. The bottom and top of each box represent the first and third quartiles; the bold line inside the box represents the median value; the whiskers represent 1.5 times the interquartile range; and points outside the whiskers represent outliers. A: gastric antrum; B: gastric body; C: lesion of intraepithelial neoplasia or cancer.

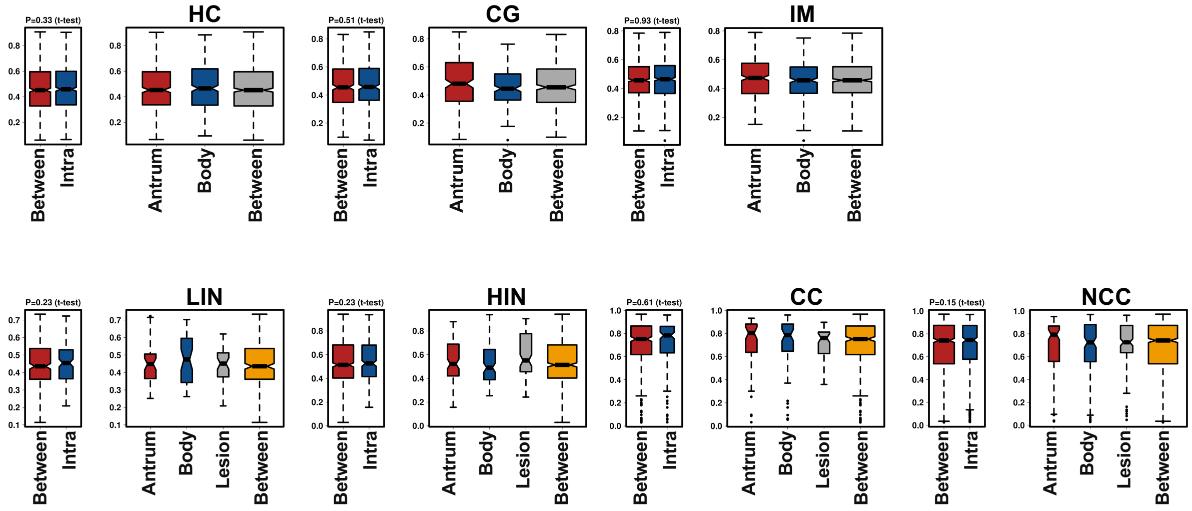


**Supplementary Figure S4**. Differences in between- and within-site Bray-Curtis distances of the gastric microbiome. Unpaired t-test was applied for all comparisons. The bottom and top of each box represent the first and third quartiles; the bold line inside the box represents the median value; the whiskers represent 1.5 times the interquartile range; and points outside the whiskers represent outliers.

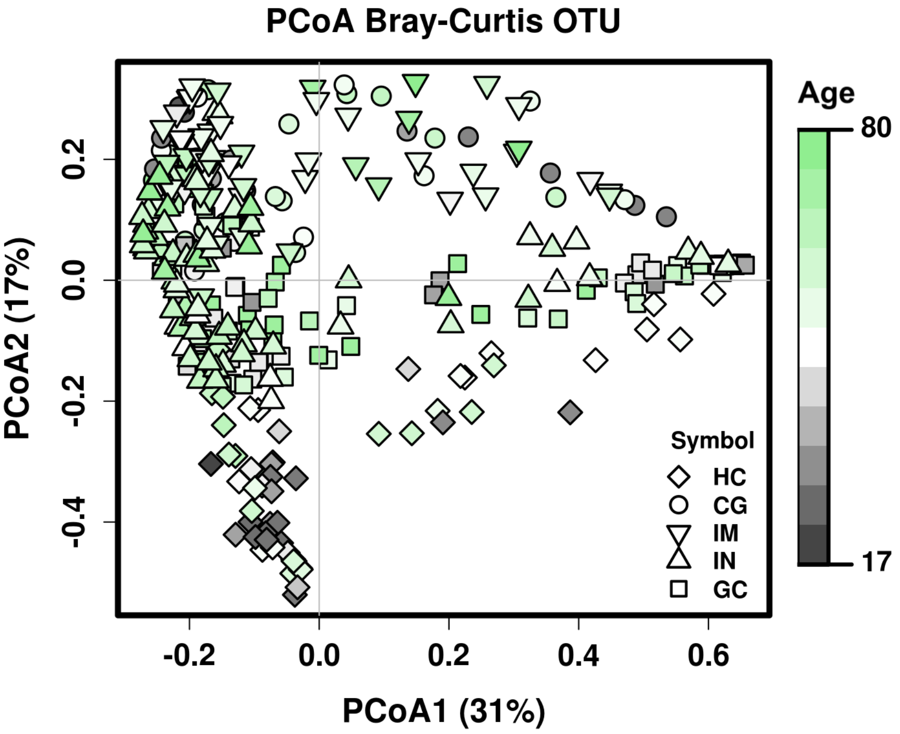


**Supplementary Figure S5.** The influence of age in the gastric microbiota composition. Bray-Curtis-based PCoA of gastric bacterial communities in the five sample groups. Each point corresponds to a sample that colored according to the age of the subject and shaped according to the gastric histological types.

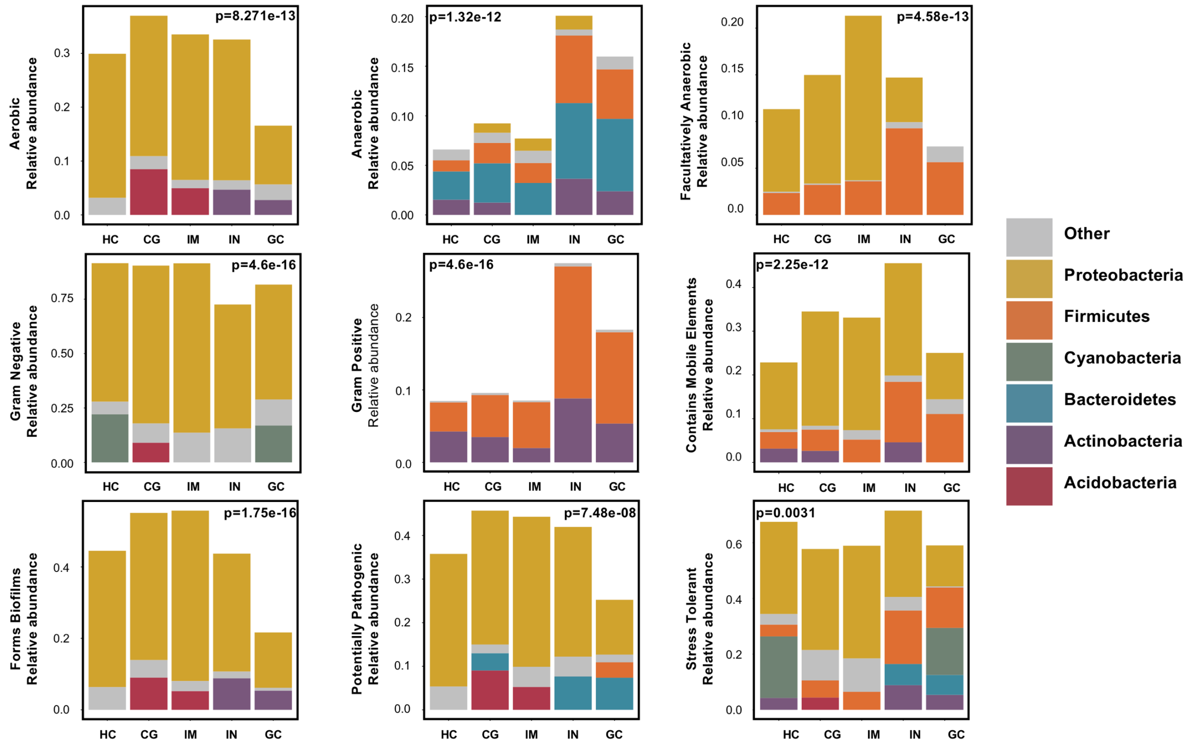


**Supplementary Figure S6.** BugBase predicts differences of organism-level microbiome phenotypes between the sample groups. The corresponding OTU contribution plots of the relative abundance of phyla possessing each phenotype are shown with bar plots. Statistical significance was assessed by Kruskal-Wallis test followed by pairwise Mann-Whitney-Wilcoxon tests (**Supplementary Table S5**).

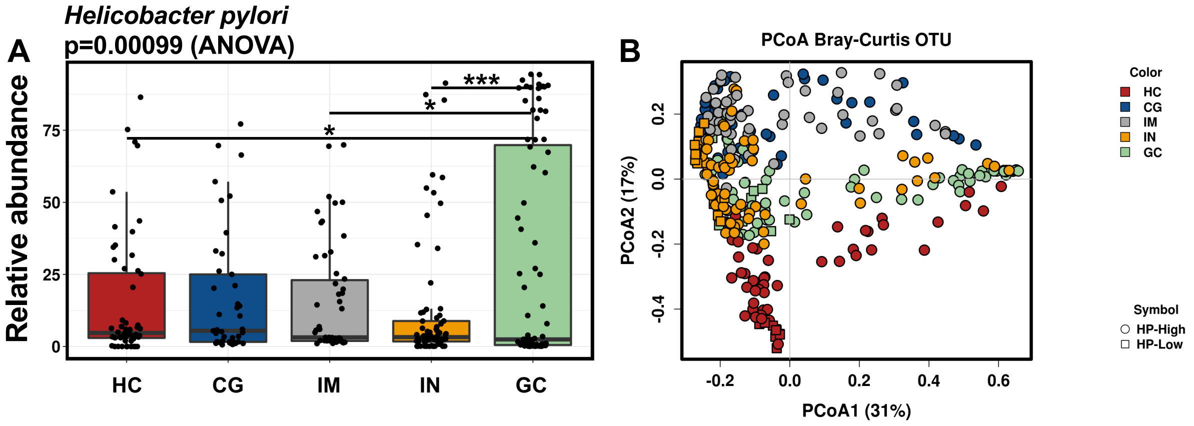


**Supplementary Figure S7.** The influence of *H. pylori* on the composition of gastric microbiota. **(A)** Relative abundance of *H. pylori* in each sample group. The bottom and top of each box represent the first and third quartiles; the bold line inside the box represents the median value; the whiskers represent 1.5 times the interquartile range; and points outside the whiskers represent outliers. Pair-wise comparisons are done by t-test and annotated as *p < 0.05, and ***p < 0.001. **(B)** Bray-Curtis-based PCoA of gastric bacterial communities in the five sample groups with indication of *H. pylori*-low and *H. pylori*-high status. Each point corresponds to a sample that colored according to the relative abundance of *H. pylori* and shaped according to the gastric histological types.

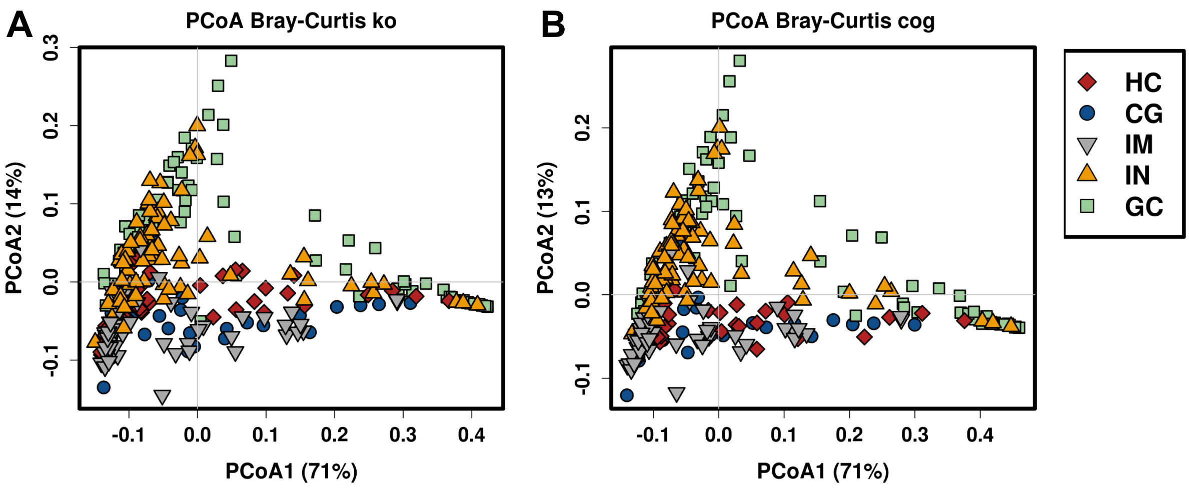


**Supplementary Figure S8.** Principal component analysis (PCA) comparing the metagenome predictions for **(A)** KO and **(B)** COG of the gastric microbiota among the six phenotypes. Samples are colored and shaped by phenotype of the gastric mucosa.

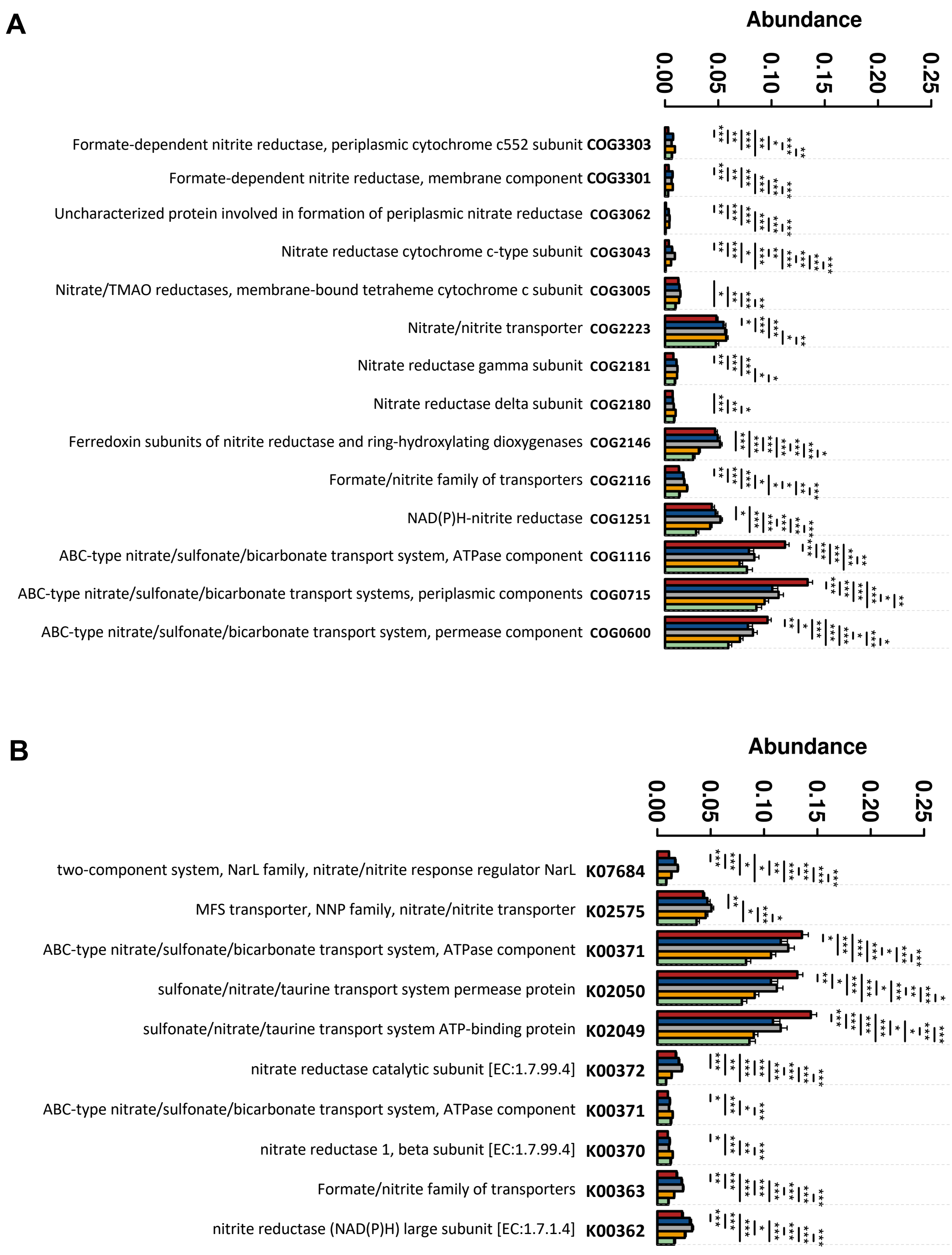


**Supplementary Figure S9.** Functional contents involved in nitrate reductase and nitrite reductase metabolism are significantly different across the gastric histological types. The normalized relative abundance of functional content of nitrate reductase and nitrite reductase in each phenotypic group predicted using **(A)** COG and **(B)** KO. Statistical significance was assessed by One-way ANOVA ($p<0.05$) followed by pairwise t-test (*$p<0.05$, **$p<0.01$ and ***$p<0.001$).
